# Mechanical Characterization and Interface Evaluation of Multi-Material Composites Manufactured by Hybrid Fused Deposition Modeling (HFDM)

**DOI:** 10.3390/polym17121631

**Published:** 2025-06-12

**Authors:** Salih Dağlı

**Affiliations:** Department of Mechanical Engineering, Sinop University, Sinop 57000, Türkiye; sdagli@sinop.edu.tr

**Keywords:** Hybrid Fused Deposition Modeling (HFDM), multi-material composites, mechanical properties, interface evaluation, additive manufacturing

## Abstract

In this study, the mechanical behavior and interfacial bonding characteristics of multi-material composites produced using the Hybrid Fused Deposition Modeling (HFDM) technique were systematically investigated. Polylactic Acid (PLA), Polyethylene Terephthalate Glycol (PETG), and Acrylonitrile Butadiene Styrene (ABS) filaments were utilized within a single structure to explore the effects of material combinations on mechanical performance. Specimens were fabricated using two distinct levels of infill density (50–100%) and raster angle (45–90°) to evaluate the influence of these parameters on tensile strength, flexural resistance, and impact toughness. Experimental tests were conducted following ASTM standards, and microstructural examinations were performed using Scanning Electron Microscopy (SEM) to assess interfacial adhesion between different polymers. The results revealed that PETG demonstrated the highest tensile strength among single-material samples, while the PLA-PETG-ABS configuration exhibited notable mechanical stability among hybrid structures. Increasing infill density and raster angle significantly enhanced mechanical performance across all configurations. SEM analyses confirmed that interfacial bonding quality critically affected structural integrity, with better adhesion observed in PLA–PETG interfaces compared to PLA–ABS transitions. The potential of HFDM in developing tailored multi-material components with optimized mechanical properties offers valuable insights for the advancement of functional additive manufacturing applications in engineering fields.

## 1. Introduction

Additive manufacturing (AM) technologies have revolutionized production methods by offering unprecedented flexibility in design, rapid prototyping capabilities, and material-efficient fabrication processes [1,2]. Among the wide array of AM techniques, Fused Deposition Modeling (FDM) has emerged as a dominant method due to its simplicity, cost-effectiveness, and compatibility with a variety of thermoplastic materials [3]. In FDM, objects are constructed layer-by-layer by extruding molten thermoplastic filaments along predefined toolpaths, resulting in parts that can be tailored in terms of geometry, internal structure, and mechanical properties [4,5].

While FDM provides several advantages, including minimal post-processing, reduced lead times, and decentralized manufacturing potential, it is traditionally limited by the use of single-material systems [6]. This constraint often restricts the mechanical performance, thermal stability, and functional versatility of printed components [7,8]. To address these limitations, multi-material additive manufacturing (MMAM) has garnered significant interest, allowing for the combination of different materials within a single build to leverage their complementary properties [9,10,11,12].

Hybrid Fused Deposition Modeling (HFDM) has emerged as a promising technique enabling the deposition of multiple filaments with distinct properties during a single printing process. This approach enables the fabrication of functionally graded structures, offering tailored mechanical, thermal, and chemical performance across different regions of a part [13,14]. In HFDM, different polymers such as Polylactic Acid (PLA), Polyethylene Terephthalate Glycol (PETG), and Acrylonitrile Butadiene Styrene (ABS) can be combined to enhance the overall structural efficiency and to create parts that meet diverse operational demands [15,16].

Each of these materials possesses unique characteristics: PLA is renowned for its high dimensional accuracy, ease of processing, and biodegradability; PETG offers superior chemical resistance, UV stability, and flexibility; while ABS is appreciated for its high impact strength and thermal stability [17,18].

The integration of multiple materials, however, introduces challenges related to interfacial adhesion, thermal mismatch, and differential shrinkage during cooling, which can compromise the mechanical integrity of printed parts [19,20]. Ensuring strong interfacial bonding between dissimilar materials remains one of the primary hurdles in achieving structurally sound multi-material composites. Factors such as printing temperature, surface energy compatibility, and cooling rates significantly influence the quality of interfacial bonding and, consequently, the mechanical performance of the final product [21,22].

Furthermore, printing parameters such as infill density (ID) and raster angle (RA) have been demonstrated to significantly affect the strength, stiffness, and durability of FDM-printed structures. RA determines the orientation of the filaments within the layers and affects the stress distribution and fracture behavior, especially under load, in relation to fiber orientation. ID directly affects the internal density of the part and therefore its load-carrying capacity. In this context, RA and ID parameters were chosen because they have significant effects on the mechanical performance of the parts produced with FDM [23,24]. Higher infill densities generally enhance load-bearing capacity by reducing internal voids, while the orientation of raster lines relative to the loading direction influences the anisotropic mechanical behavior of the printed parts [25]. These relationships necessitate a comprehensive understanding of how process parameters interact with material properties, particularly in the context of multi-material fabrication.

Several studies have addressed the mechanical characterization of individual filaments and simple two-material combinations. For example, Ahmed et al. [26] investigated carbon-fiber-reinforced PLA and ABS composites, reporting enhanced tensile properties compared to single-material components. Tamburrino et al. [27] explored multi-material printing using thermoplastic polyurethane (TPU), PLA, and chlorinated polyethylene (CPE), emphasizing the importance of interfacial bonding. Similarly, Shi et al. [21] evaluated blended and laminated multi-material structures, revealing that laminated configurations exhibited superior tensile strength due to improved interlayer adhesion. However, most previous works have focused on either binary material systems or homogeneous filament blends, leaving a substantial gap in understanding the behavior of more complex hybrid structures comprising three distinct materials.

Moreover, the morphological evolution at material interfaces during mechanical loading remains an underexplored area. Scanning Electron Microscopy (SEM) analyses have proven invaluable for investigating fracture surfaces, revealing crucial information about crack propagation, layer delamination, and the nature of failure mechanisms [28,29]. Hence, complementing mechanical testing with microstructural characterization is essential to fully elucidate the performance of HFDM-fabricated multi-material composites.

Within this context, the present study aims to systematically investigate the mechanical performance and interfacial quality of PLA, PETG, and ABS composites fabricated using the HFDM process. Test specimens were designed and manufactured using two distinct levels of ID (50–100%) and RA (45–90°) to assess their influence on tensile, flexural, and impact properties. The fabrication process employed an Anycubic Kobra Combo 3D printer equipped with multi-material printing capabilities. Printing parameters including nozzle diameter, layer thickness, cooling speed, and surface pattern were carefully optimized based on the characteristics of the selected filaments.

Mechanical testing was conducted according to relevant ASTM standards: ASTM D638-1 [30] for tensile testing, ASTM D5045-14 [31] for three-point bending, and ASTM D256-04 [32] for impact strength evaluation. Additionally, SEM analyses were performed on fractured surfaces to observe interfacial bonding quality and failure mechanisms.

By providing a detailed mechanical and microstructural characterization of HFDM-fabricated multi-material composites, this study contributes valuable insights into the process–structure–property relationships governing MMAM. The outcomes are expected to inform the optimization of design and processing strategies for developing high-performance functional parts in applications demanding tailored mechanical behaviors and enhanced structural reliability.

## 2. Materials and Methods

### 2.1. Materials

Three different thermoplastic filaments (natural without coloring additives)—Polylactic Acid (PLA), Polyethylene Terephthalate Glycol (PETG), and Acrylonitrile Butadiene Styrene (ABS)—were selected for fabricating multi-material composites using the Hybrid Fused Deposition Modeling (HFDM) method. These materials were chosen based on their distinct mechanical and chemical properties, enabling a comprehensive assessment of the interfacial and mechanical behavior in hybrid structures. The technical properties of the filaments, as provided by the manufacturer (Anycubic, Guangdong, China), are summarized in Table 1.

To ensure optimal processing conditions, nozzle temperatures, bed temperatures, and cooling speeds were carefully adjusted for each filament type. The specific values were selected to promote sufficient interlayer adhesion while minimizing thermal distortions during printing.

### 2.2. Specimen Preparation

**Figure 1:**
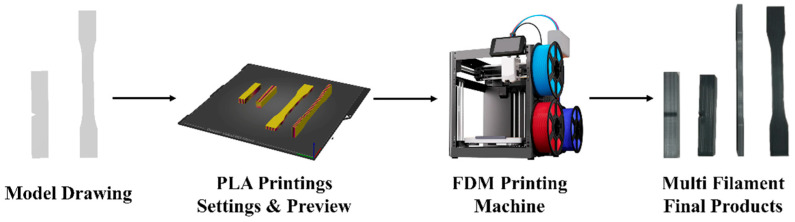
HFDM printing process illustration.

The specimens for tensile, flexural, and impact testing were designed using SolidWorks CAD software (2024 SP5) and sliced with Anycubic Slicer (v1.4.4) to generate G-code instructions for multi-material printing. The fabrication was carried out using an Anycubic Kobra Combo 3D printer (Shenzhen Anycubic Technology Co., Ltd., Guangdong, China) equipped with a multi-material printing module. An illustration of the HFDM printing process is given in Figure 1.

Specimens were printed using a 1.75 mm filament diameter and a 0.4 mm nozzle size. The layer thickness was fixed at 0.2 mm, and the surface pattern was configured in a linear mode to ensure consistent mechanical performance across different samples. RAs were varied between 45° and 90° and IDs were set at 50% and 100% to study their effects on mechanical behavior. Detailed printing parameters are listed in Table 2.

**Figure 2:**
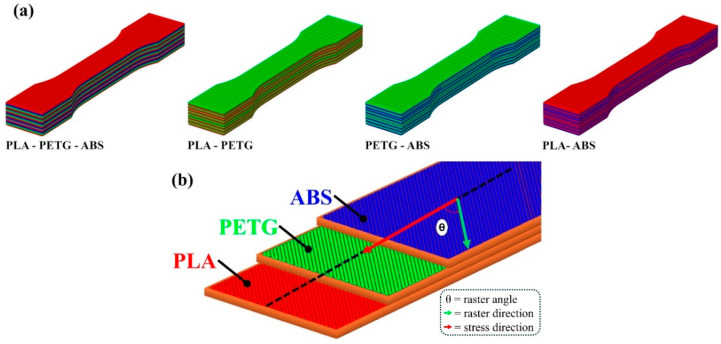
(**a**) Multi-material manufacturing configurations, (**b**) part raster angle and stress directions.

Moreover, different multi-material configurations were developed by combining PLA, PETG, and ABS filaments in various sequences. Each configuration was assigned a unique specimen code, as outlined in Table 3. An illustration showing the multi-material configurations and the printing orientations is presented in Figure 2, providing a visual overview of how material transitions and raster patterns were arranged during the fabrication process. Each specimen was fabricated with 30 layers, ensuring a uniform total thickness across all test samples.

### 2.3. Mechanical Testing

#### 2.3.1. Tensile Testing

Tensile tests were performed according to ASTM D638-1 standards using a Shimadzu EHF-LV005K2-010 (Kyoto, Japan) universal testing machine. A constant crosshead speed of 5 mm/min was applied at room temperature. For each configuration, three identical specimens were tested to ensure the repeatability of results. The ultimate tensile strength (UTS), elastic modulus, and elongation at break were recorded. Stress–strain curves were generated for further comparative analysis among different material combinations and printing parameters.

#### 2.3.2. Flexural Testing

Single-edge notched bending (SENB) tests were conducted in accordance with ASTM D5045-14 standards using a Shimadzu (Kyoto, Japan) three-point bending fixture. The support span was set to 48 mm, and the crosshead speed was maintained at 5 mm/min. Again, three specimens per configuration were tested to ensure data reliability. Flexural strength, flexural modulus, and strain at fracture were extracted from the force–displacement curves.

#### 2.3.3. Impact Testing

Impact resistance was evaluated using notched Charpy specimens following ASTM D256-04 standards. Tests were performed on a Utest UTCI-0450J (Ankara, Turkey) impact testing machine operating at an impact speed of 5.4 m/s. Energy absorption during fracture was measured and recorded for each configuration to assess the toughness of the printed composites.

### 2.4. Microstructural Characterization

SEM analyses were performed using a JEOL JCM-7001F (Tokyo, Japan) microscope to observe the fractured surfaces of the tensile, flexural, and impact specimens. Prior to imaging, specimens were sputter-coated with a thin layer of gold (approximately 2–3 μm) to prevent charging and enhance image quality. Micrographs focused on interfacial regions between different materials, allowing for the evaluation of interlayer bonding, crack propagation patterns, and the presence of voids or delaminations. These observations were critical for interpreting the mechanical behavior in relation to the structural integrity of the multi-material composites.

## 3. Results and Discussion

### 3.1. Tensile Test Results

**Figure 3:**
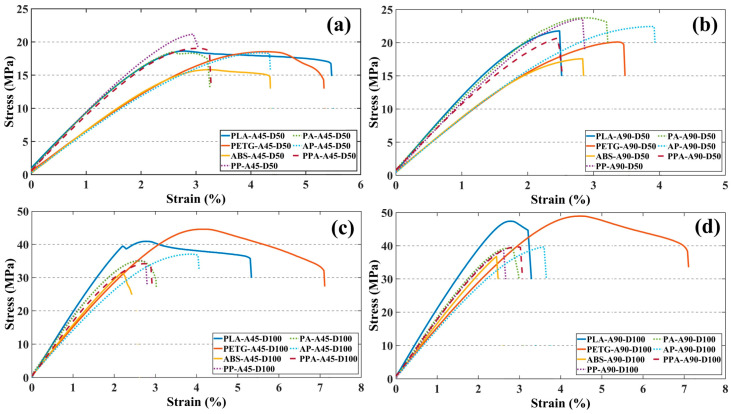
Stress–strain curves of tensile test specimens: (**a**) ID 50% RA 45°, (**b**) ID 50% RA 90°, (**c**) ID 100% RA 45°, (**d**) ID 100% RA 90°.

The stress–strain results obtained from the tensile test of the specimens are shown in Figure 3.

When the mechanical properties of the tensile specimens are compared, it can be seen that the specimens produced using PLA and PETG filaments have the highest mechanical performance. Among the single material specimens, the PETG-A90-D100 tensile specimen has the highest tensile strength of 48.94 MPa and the highest strain value of 4.45%. PLA-A90-D100 showed similar mechanical performance to PETG-A90-D100 with a tensile strength of 47.40 MPa and strain value of 2.77%. PLA was found to have limited ductility with a lower strain of approximately 37.75% compared to PETG. The mechanical properties of pure ABS parts proved worse than those of PLA and PETG in every specimen. The high thermal shrinkage tendency and low bond surface energy of ABS caused the structure to become brittle and reduced its mechanical performance at reduced ID and angular directional stresses [33,34]. As a result of the tensile test, the maximum tensile strength values of all specimens are given in Table 4.

When comparing the mechanical performance of specimens made from multi-material hybrid composite, it can be seen that different filament combinations produce significant differences in tensile strength and strain behavior. Among the multi-material hybrid specimens, the highest tensile strength was determined as 39.65 MPa for PPA-A90-D100 and AP-A90-D100 specimens. It was observed that the tensile strength of PA-A90-D100 was 39.29 MPa. The tensile strength values of the multi-material hybrid structures were very close to each other. The high ductility of PETG and the stiffness of ABS create a balanced structure. Although the contribution of PLA provided high elasticity and stiffness, the increase in strength was limited in several combinations due to interfacial incompatibility [35]. When the multi- and single-material specimens were compared, it was determined that PPA-A90-D100 had 18% lower tensile strength than PETG-A90-D100. The main reasons for the decrease in tensile strength in hybrid structures can be attributed to incompatibility between materials with different properties and bond weaknesses due to different thermomechanical expansion behaviors. In multi-material structures, adhesion problems that occur between the layers during printing lead to stress accumulation between the layers and reduce the load-carrying capacity [36].

When the unit elongation values were analyzed, a significant increase in ductility was observed in the multi-material structures between the multi-material specimens and the base PLA and ABS specimens. Specifically, AP-A45-D50 shows 4.13% strain, which is 49.6% and 29% higher than PLA-A45-D50 and ABS-A45-D50 respectively. The ductile structure and high deformation capacity of PETG contributed to an increase in the ductility in multi-material structures [37,38].

**Figure 4:**
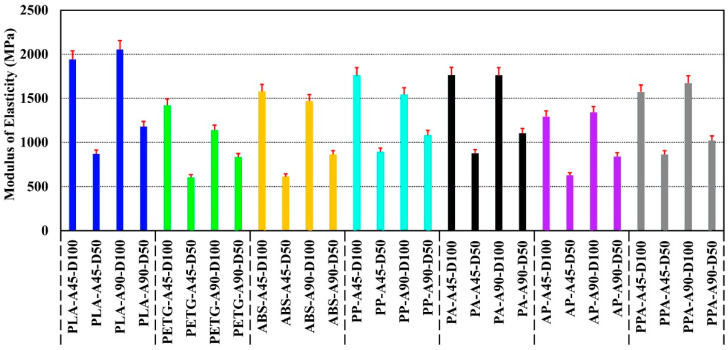
Average modulus of elasticity values of the tensile specimens.

The modulus of elasticity of the specimen was calculated from the stress–strain values obtained in the tensile test. The average values of the results obtained are shown in Figure 4.

**Figure 5:**
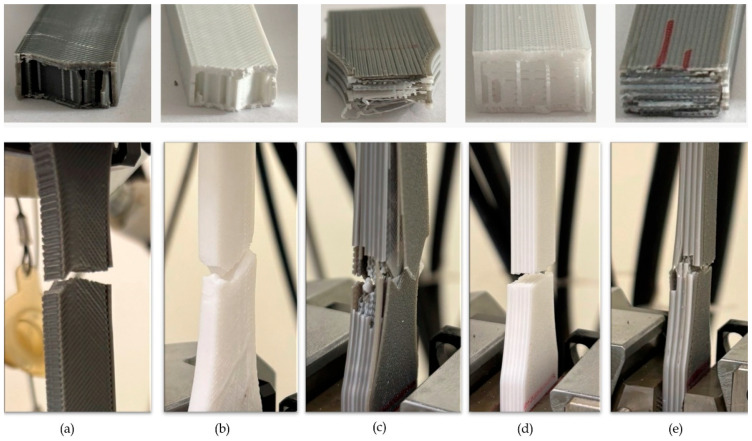
Fracture details of the specimens in the tensile test: (**a**) PLA-A45-D50, (**b**) PETG-A45-D50, (**c**) PPA-A90-D100, (**d**) PP-A45-D50, (**e**) AP-A90-D100.

The modulus of elasticity values of the specimens produced from PLA, PETG, and ABS filaments showed significant differences depending on the material type [39,40,41]. PLA-A90-D100 offered the highest rigidity with a modulus of elasticity of 2054 MPa, about 80% higher than PETG-A90-D100 and 40% higher than ABS-A90-D100. Compared to PLA-A90-D100, PLA-A45-D50 was found to have an elastic modulus value approximately 57% lower due to the effect of ID and RA on the elastic behavior. PETG had a 40% lower elastic rigidity due to its irregular structure but offered an advantage in terms of deformability. When the multi-material hybrid specimens were analyzed, PA-A90-D100 showed only a 14% reduction in modulus compared to PLA-A90-D100, while maintaining its stiffness. On the other hand, AP-A90-D100 showed a 35% reduction in modulus of elasticity and increased flexibility. Although PPA-A90-D100 is sufficient in terms of rigidity with a modulus of elasticity value of 1673 MPa, the incompatibility between the layers due to printing temperature differences between PLA and ABS caused interfacial weaknesses in the structure (Figure 5) [23,42].

ID and RA performed a critical role in the tensile properties of both single and multi-material specimens. Specimens with ID 50% and RA 45° parameters showed mechanical performance losses of up to 70% compared to specimens with ID 100% and RA 90° parameters. PPA-A90-D100 was found to have a 52% higher tensile strength value than PPA-A45-D50. By increasing ID, the voids in the material are reduced and structural integrity is achieved throughout the parts. In addition, the fact that the compression angle is in the same direction as the tensile direction increased the strength and stiffness values of the materials. In this case, the RA and ID parameters were found to be the determining factors in the tensile strength and elongation at the break of the specimens.

When the results were evaluated, the tensile strength values of the multi-material hybrid specimens were approximately 18% lower than the pure specimens, but the hybrid specimens provided more than a 30% increase in strain capacity. Thus, the multi-material hybrid composites showed a very successful mechanical performance in terms of brittleness–ductility balance. It was also revealed that ID and RA, in addition to the filament type used, play a significant role in the mechanical behavior of the structures [43]. With the right material compatibility and printing parameter optimization, multi-material structures can offer significant advantages for engineering designs in terms of both stiffness and deformation capacity.

### 3.2. Flexural Test Results

**Figure 6:**
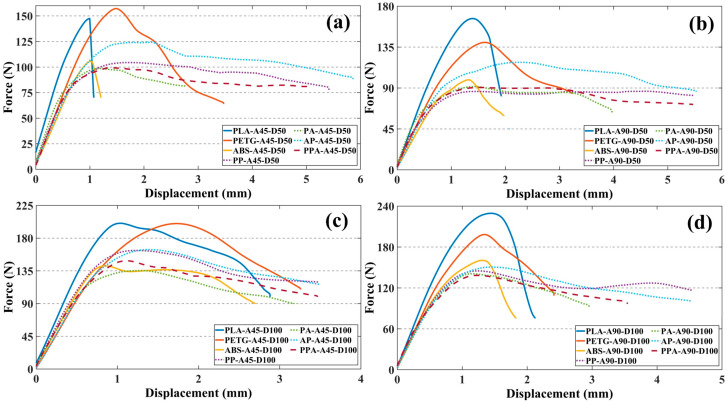
Force–displacement curves of SENB specimens: (**a**) ID 50% RA 45°, (**b**) ID 50% RA 90°, (**c**) ID 100% RA 45°, (**d**) ID 100% RA 90°.

The force–displacement results obtained from the SENB test of the specimens are shown in Figure 6.

When the results of the SENB test were examined, it was found that the specimens containing PLA had the highest bending strength. The PLA-A90-D100 specimen exhibited the highest bending strength of 230.49 N, 15% higher than PETG-A90-D100 and 42% higher than ABS-A90-D100. However, this high bending strength of PLA was limited by its low deformation capacity and showed a brittle fracture behavior with increasing load. In contrast, PETG and ABS specimens, while having lower flexural strength, showed a more ductile behavior with increasing deformation time.

**Figure 7:**
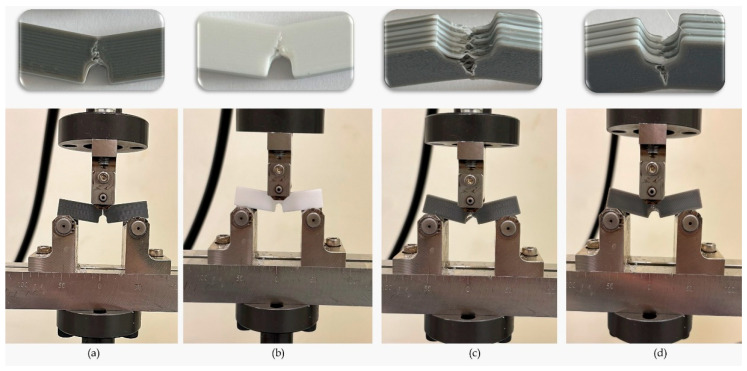
Fracture details of the specimens in SENB test: (**a**) PLA-A90-D100, (**b**) PET95, (**c**) PPA-A90-D100, (**d**) AP-A90-D100.

Multi-material hybrid specimens exhibited a more balanced mechanical behavior profile. In specimens where materials such as PLA, PETG, and ABS were used together, a balance between rigidity and ductility was observed depending on the combination of layers within the structure (Figure 7). Although the PPA-A45-D100 specimen had 22% lower flexural strength than PLA-A45-D100, it was able to support the load for a longer period after fracture and exhibited softer mechanical behavior. Similarly, PA-A90-D100 showed a lower strength of about 28% compared to PLA-A90-D100 but showed tougher behavior with an increase in deformation capacity of about 80%. These results show that multi-material structures exhibit a more stable mechanical performance underload due to interlayer interaction. In addition, it can also be stated that multi-material structures delay crack propagation and increase fracture toughness [44].

**Figure 8:**
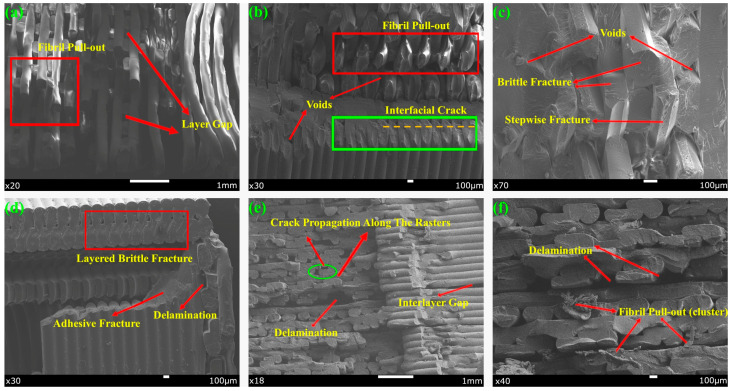
SEM images of the fracture surfaces of the specimens after SENB test: (**a**) PPA-A45-D50, (**b**) AP-A45-D100, (**c**) AP-A45-D100, (**d**) PLA-A90-D50, (**e**) PPA-A90-D100, (**f**) PPA-A90-D100.

SEM images of the fracture zones of the three-point bending test specimens at different zoom-ins are given in Figure 8.

When the SEM images obtained from the fracture regions of the specimens after the SENB were examined, it was found that the use of multiple materials resulted in the formation of complex structures on the fracture surfaces and the formation of multimodal fracture types.

The fracture morphology observed on PLA-A90-D50 exhibited typical layered brittle fracture characteristics. The prominent delamination and adhesion-induced fractures along the interfaces are consistent with the literature suggesting that PLA exhibits brittle behavior due to low interlayer adhesion [24]. In the PLA-A90-D50 specimen, fracture lines running parallel to the print layers indicate that the production parameters (high infill density, perpendicular print angle) have a significant effect on the fracture morphology [45].

In contrast, more complex deformation mechanisms were observed at the fracture surfaces in samples containing multiple filaments such as PPA-A45-D50 and AP-A45-D100. In particular, the PPA-A45-D50 specimen exhibited features such as fibril pull-out and layer gaps, and it was determined that these formations were caused by incompatibility between the layers of materials with different thermal stress properties, such as PETG and ABS [46]. In the AP-A45-D100 specimen structure, interfacial crack propagation and void formations were evident, and it was observed that fracture propagation was facilitated due to the weak interfacial bonding forces of different filaments. Such interfacial defects in multi-material structures cause loss of mechanical performance in parts [47,48].

When the ID effect was evaluated on a material basis, it was observed that large void formation and delamination events occurred more intensely in PPA-A90-D100 and PLA-based specimens. In PPA-A90-D100, crack propagation along the craters was observed. Reduced ID leads to weakening of the joints between the layers, allowing cracks to propagate easily through the layers [49]. It was also found that the weak zones formed under load in multi-material structures with reduced ID caused fracture initiation.

When analyzing the effect of RA, step fracture-type fractures were observed intensively, especially in AP-A45-D100. Such fractures are due to stress concentrations caused by the intersection of the layer orientation with the direction of loading. On the other hand, PLA-A90-D50 showed smoother fracture surfaces as the load application direction and print layers were aligned [50].

### 3.3. Impact Test Results

**Figure 9:**
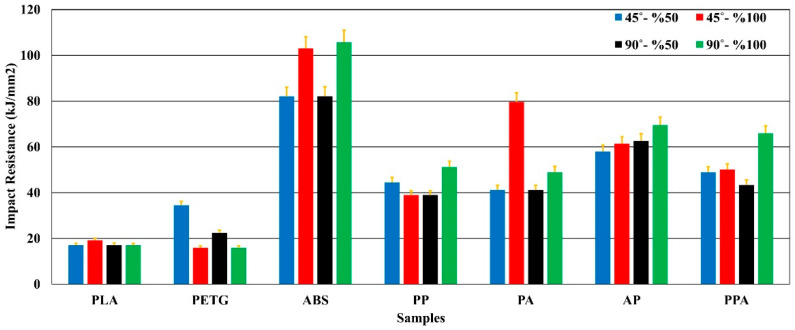
Impact strength values of test specimens.

The impact resistance results obtained from the notch impact test applied to the produced specimens are shown in Figure 9.

As a result of the notch impact tests, it was found that the multi-material hybrid specimens generally exhibited higher mechanical performance in terms of impact strength than single-material structures. Among the single-material specimens, the highest impact strength of ABS-A90-D100 reached 105.73 kJ/mm^2^. This value is 520% higher than the impact strength of PLA-A90-D100 of 17.05 kJ/mm^2^. This difference can be explained by the ductile structure of ABS and its internal structure that can absorb crack propagation [51]. The rigidity and low energy absorption capacity of PLA caused it to exhibit brittle behavior under impact [52,53].

**Figure 10:**
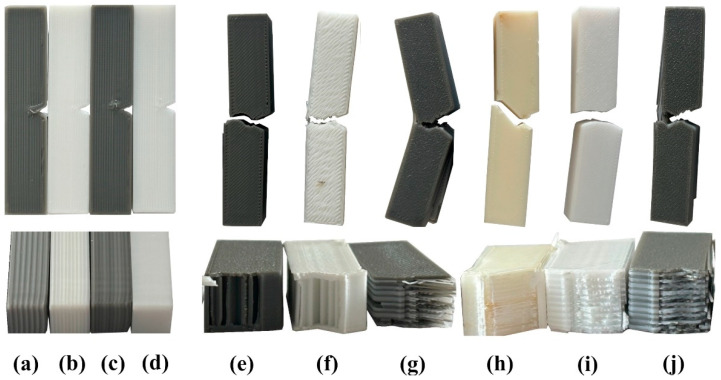
Fracture details of the specimens after notch impact test: (**a**) PP-A90-D100, (**b**) AP-A90-D100, (**c**) PPA-A90-D50, (**d**) PLA-A90-D100. (**e**) AP-A45-D50, (**f**) PETG-A45-D50, (**g**) PPA-A45-D50, (**h**) PETG-A45-D50, (**i**) ABS-A45-D50, (**j**) AP-A45-D50.

When the multi-material structures were examined, important results were obtained in terms of impact performance. In particular, PPA-A90-D100, with an impact energy of 65.95 kJ/mm^2^, exhibited approximately 286% more strength than PLA-A90-D100 and 313% more strength than PETG-A90-D100. This increase can be considered a result of the combination of different polymers combining rigidity (PLA), impact damping ability (PETG), and ductility (ABS) in one structure [28,54]. The AP-A90-D100 specimen achieved 69.55 kJ/mm^2^, 310% better impact strength than PLA-A90-D100. This is due to the more flexible but non-uniform structure of PETG, which increases its ability to dissipate fracture energy by increasing the adhesion between the layers in ABS (Figure 10). The impact strength of AP-A90-D100 was 35.76% higher than that of PP-A90-D100. This shows that the presence of ABS in multi-material structures is extremely important in terms of impact resistance [55].

When the effect of the printing parameters was evaluated, it was determined that the ID and RA parameters also had significant effects in terms of impact strength. In general, ID 100% and RA 90° parameters provided approximately 35% to 60% higher impact strength than ID 50% and RA 45° parameters. PPA-A90-D100 showed 51.95% higher impact strength performance than PPA-A90-D50 and 34.81% higher than PPA-A45-D50. This is due to the more uniform alignment of the interlayer bonds and the reduction of the material internal void, resulting in resistance to impact [56,57].

**Figure 11:**
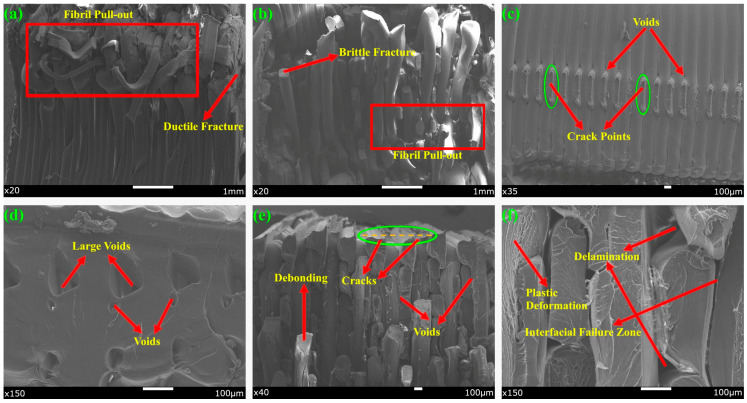
SEM images of the fracture surfaces of the specimens after notch impact test: (**a**) AP-A45-D100, (**b**) PPA-A45-D50, (**c**) PLA-A90-D50, (**d**) PLA-A90-D50, (**e**) PPA-A90-D100, (**f**) PPA-A90-D100.

The results of the SEM analysis of the fracture zones of the specimens at different zoom levels as a result of the notched impact test are shown in Figure 11.

SEM images obtained after the notch impact test revealed that the fracture behavior of the specimens containing single and multiple filament combinations differed significantly. These differences are due to the mechanical properties of the material types used and interfacial interactions between the materials. Fibril pull-out regions and ductile fracture characteristics were observed in the AP-A45-D100 specimen. Similar (fibril pull-out) and brittle fracture structures were observed in PPA-A45-D50. The amorphous and ductile structure of PETG tends to dissipate energy by plastic deformation under impact, which causes fibrils to pull out and break at the fracture line [58]. At the same time, the more rigid structure of PLA accelerated crack propagation in some areas of the fracture surface.

The fracture surface of PPA-A45-D50 exhibited hybrid fracture modes including both brittle fracture and fibril pull-out. Multi-material systems of this nature created stress concentrations, especially between the tougher behavior of PETG and ABS and the brittleness of PLA, leading to a change in the direction of crack propagation and energy dissipation in different ways [28]. The distribution of different materials along the layers creates mechanical disharmony stresses, which in turn trigger interfacial failure zones.

In the PLA-A90-D50 specimen, crack initiation tendencies (crack points) and significant voids were observed especially at the notch initiation points. At this point, fractures were sudden and crack-oriented due to the weak impact toughness of PLA. It was determined that the large voids seen in PLA-A90-D50 indicate that the material did not form sufficient interlayer fusion during printing and these areas caused fracture initiation. Multimodal fracture types such as debonding, delamination, voids, plastic deformation, and interfacial failure zone were prominent in PPA-A90-D100. Separation zones between layers in the material structure are indicative of inter-filament adhesion problems. The low surface energy of ABS in particular favored the formation of delamination by making it difficult to fully fuse with other filaments. At the same time, the plastic deformation zones formed with the addition of PETG indicate that some of the impact energy was absorbed by permanent deformation instead of elastic deformation [58].

It was found that delamination events occurred more frequently at the interfaces and crack propagation changed direction at the material boundaries when using multiple materials. However, it was found that the multi-material structure significantly alters the fracture behavior due to interlayer structural disorder, independent of ID and RA effects. When material combinations were not selected correctly, fractures were found to initiate mostly at the interface, reducing the mechanical integrity of the parts.

### 3.4. Microstructural Analysis

**Figure 12:**
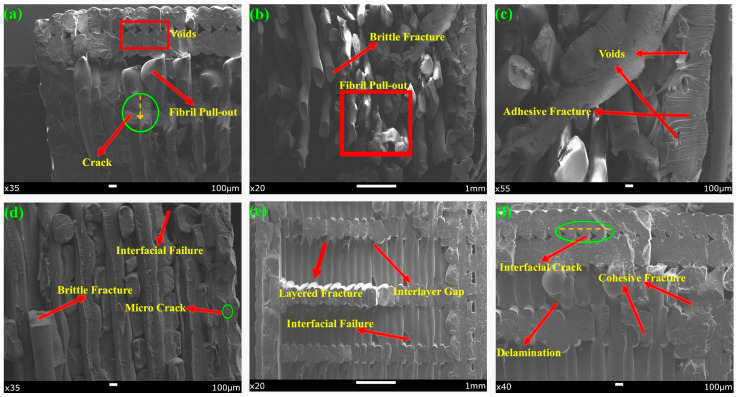
SEM images of the fracture surfaces of the specimens after the tensile test: (**a**) AP-A45-D100, (**b**) PPA-A45-D50, (**c**) PPA-A45-D50, (**d**) PPA-A90-D100, (**e**) PLA-A90-D50, (**f**) PLA-A90-D50.

The results of the SEM analysis of the fracture zones of the specimens at different zoom levels as a result of the tensile test are shown in Figure 12.

Multimodal fracture structures such as fibril pull-out, microcrack formation, interlayer gap, and interfacial failure were observed especially on the fracture surfaces of multi-material hybrid composites. Significant fibril pull-out, voids, and crack propagation were observed on the fracture surface of AP-A45-D100. While the ductile nature of PETG favors fibril formation by plastic deformation, the more brittle nature of PLA caused cracks to propagate easily in the regions where these fibrils formed [36]. This is due to mechanical incompatibilities caused by the combination of filaments with different thermo-mechanical properties [20].

In the PPA-A45-D50 specimen, brittle fracture regions and adhesive fracture caused by fibril pull-out and adhesion weaknesses were observed. During 3D printing, void formations occurred at the fracture zone interfaces due to the incompatible melting behavior of filaments with different properties [59]. Such voids cause weakening of the structures under load by microcracks and reduce fracture toughness. In specimens with RA 45°, due to the angular orientation of the filaments, shear stresses increase at interfaces that are not perpendicular to the stress direction, which accelerates the formation of delaminations.

Interfacial failure, micro crack, and brittle fracture behaviors were observed together between the layers in PPA-A90-D100. Especially, the low surface energy of ABS and the viscoelastic nature of PETG facilitate the formation of microcracks by weakening the interfacial adhesion when used with PLA [24]. In addition, the ID 100% of this structure caused the deformation under load to concentrate at the interfaces and caused notch-effect fractures. PLA-A90-D50 exhibited characteristic layered fracture, interlayer gap, interfacial crack, delamination, and cohesive fracture behaviors. In structures with ID 100% and RA 90°, the filament alignment parallel to the load direction resulted in uniform but abrupt fractures at the fracture surfaces [52].

## 4. Conclusions

In this study, the mechanical properties and interfacial characteristics of multi-material composites fabricated via Hybrid Fused Deposition Modeling (HFDM) were systematically investigated. By employing PLA, PETG, and ABS filaments in various configurations and modifying printing parameters such as infill density and raster angle, the effects of material selection and processing conditions on tensile, flexural, and impact behaviors were comprehensively analyzed. Key findings from the experimental and microstructural evaluations are summarized as follows:

Material Selection: Among single-material specimens, PETG exhibited superior tensile and flexural performance compared to PLA and ABS, while ABS demonstrated the highest impact resistance. Multi-material composites, particularly those incorporating PLA–PETG–ABS configurations, provided a balanced combination of strength, toughness, and flexibility.

Infill Density: Increasing the infill density from 50% to 100% significantly enhanced the mechanical performance across all tests. Denser internal structures promoted improved load transfer, reduced internal voids, and delayed crack initiation.

Raster Angle: Specimens printed at 90° raster orientation showed higher tensile and flexural strengths due to better filament alignment with loading directions, while 45° raster orientation improved toughness and impact energy absorption through enhanced filament interlocking.

Interfacial Bonding: SEM analysis revealed that the quality of bonding at material interfaces critically influenced the overall mechanical performance. PLA–PETG interfaces exhibited stronger adhesion and more cohesive failure modes compared to PLA–ABS transitions, where delamination and void formation were prevalent.

Failure Mechanisms: Crack propagation patterns were highly dependent on the material combination and printing parameters. Multi-material specimens with optimized interfaces demonstrated crack deflection and energy dissipation mechanisms, contributing to enhanced structural reliability.

Overall, this study highlights the potential of HFDM technology in tailoring the mechanical properties of 3D-printed components through strategic material selection and careful process optimization. The findings offer valuable guidelines for the design and manufacturing of functionally graded structures in advanced engineering applications, including automotive, aerospace, and biomedical sectors.

Further research is recommended to explore the integration of reinforced or nano-enhanced filaments into HFDM multi-material structures. Additionally, the application of surface treatment techniques at material interfaces could be investigated to further improve interfacial bonding and overall mechanical performance under complex loading conditions.

## Figures and Tables

**Table 1:** Technical specifications of the filaments.

Feature	PLA	PETG	ABS
Print temperature (°C)	220	235	235
Bed temperature (°C)	60	80	80
Density (g/cm^3^)	1.23	1.27	1.06
Tensile strength (MPa)	61	52.2	40
Flexural strength (MPa)	40.7	58.1	68
Elongation at break (%)	42.5	83	30
Hardness (D-Shore)	80	71	75

**Table 2:** Printing parameters.

Properties	Parameter
Filament diameter (mm)	1.75
Nozzle diameter (mm)	0.4
Layer Thickness (mm)	0.2
Raster angle (°)	45–90
İnfill ratio (%)	50–100
Cooling speed (%)	PLA = 98, PETG = 50, ABS = 0
Surface pattern	Line

**Table 3:** Specimen configurations and sample codes.

Material	Raster Angle (°)	Infill Density (%)	Sample Code
PLA-PETG	45	50	PP-A45-D50
		100	PP-A45-D100
	90	50	PP-A90-D50
		100	PP-A90-D100
PLA-ABS	45	50	PA-A45-D50
		100	PA-A45-D100
	90	50	PA-A90-D50
		100	PA-A90-D100
ABS-PETG	45	50	AP-A45-D50
		100	AP-A45-D100
	90	50	AP-A90-D50
		100	AP-A90-D100
PLA-PETG-ABS	45	50	PPA-A45-D50
		100	PPA-A45-D100
	90	50	PPA-A90-D50
		100	PPA-A90-D100

**Table 4:** Maximum tensile strength values of the specimens (MPa).

Code	45°–%50	45°–%100	90°–%50	90°–%100
PLA	18.56	40.93	21.76	47.40
PETG	18.53	44.58	20.10	48.94
ABS	15.82	31.53	17.57	36.95
PP	21.20	35.10	23.67	38.64
PA	18.58	34.96	23.74	39.29
AP	18.36	37.05	22.44	39.65
PPA	19.03	34.19	20.68	39.64

## Data Availability

Data are contained within this article.
